# Human Calmodulin Methyltransferase: Expression, Activity on Calmodulin, and Hsp90 Dependence

**DOI:** 10.1371/journal.pone.0052425

**Published:** 2012-12-20

**Authors:** Sophia Magen, Roberta Magnani, Sitvanit Haziza, Eli Hershkovitz, Robert Houtz, Franca Cambi, Ruti Parvari

**Affiliations:** 1 Shraga Segal Department of Microbiology, Virology and Genetics, Faculty of Health Sciences, Ben Gurion University of the Negev, Beer Sheva, Israel; 2 Department of Horticulture, University of Kentucky, Lexington, Kentucky, United States of America; 3 Pediatric Endocrinology & Metabolism Unit, Soroka Medical Center, Beer Sheva, Israel; 4 Department of Neurology, University of Kentucky, Lexington, Kentucky, United States of America; 5 National Institute of Biotechnology in the Negev, Ben Gurion University of the Negev, Beer Sheva, Israel; University of Iowa, United States of America

## Abstract

Deletion of the first exon of calmodulin-lysine N-methyltransferase (*CaM KMT*, previously *C2orf34*) has been reported in two multigene deletion syndromes, but additional studies on the gene have not been reported. Here we show that in the cells from 2p21 deletion patients the loss of CaM KMT expression results in accumulation of hypomethylated calmodulin compared to normal controls, suggesting that CaM KMT is essential for calmodulin methylation and there are no compensatory mechanisms for CaM methylation in humans. We have further studied the expression of this gene at the transcript and protein levels. We have identified 2 additional transcripts in cells of the 2p21 deletion syndrome patients that start from alternative exons positioned outside the deletion region. One of them starts in the 2^nd^ known exon, the other in a novel exon. The transcript starting from the novel exon was also identified in a variety of tissues from normal individuals. These new transcripts are not expected to produce proteins. Immunofluorescent localization of tagged CaM KMT in HeLa cells indicates that it is present in both the cytoplasm and nucleus of cells whereas the short isoform is localized to the Golgi apparatus. Using Western blot analysis we show that the CaM KMT protein is broadly expressed in mouse tissues. Finally we demonstrate that the CaM KMT interacts with the middle portion of the Hsp90 molecular chaperon and is probably a client protein since it is degraded upon treatment of cells with the Hsp90 inhibitor geldanamycin. These findings suggest that the CaM KMT is the major, possibly the single, methyltransferase of calmodulin in human cells with a wide tissue distribution and is a novel Hsp90 client protein. Thus our data provides basic information for a gene potentially contributing to the patient phenotype of two contiguous gene deletion syndromes.

## Introduction

CaM KMT (previously C2orf34) has been reported to be within the deletion region of two autosomal, recessive syndromes. The first reported, 2p21 deletion syndrome is caused by a homozygous deletion of 179,311 bp on chromosome 2p21, which includes the type I cystinuria gene (SLC3A1), the protein phosphatase 2Cβ gene (PPMB1), prolylendopeptidase like (PREPL) gene, and the first exon of the CaM KMT gene. Patients homozygous for this deletion present with cystinuria, neonatal seizures, hypotonia, severe mental and growth retardation, facial dysmorphism and reduced activity of all, except the 2^nd^ mitochondrial encoded respiratory chain enzymatic complexes [1], [2]. The second disorder is atypical hypotonia-cystinuria syndrome (HCS) caused by a smaller deletion of 77.4 kb on the 2p21 chromosome that encompasses SLC3A1, PREPL and similarly to the first report the first exon of CaM KMT. Atypical HCS patients present with a phenotype partly similar to 2p21 deletion syndrome such as severe hypotonia at birth, poor feeding, facial dysmorphism, growth retardation and cystinuria but with growth hormone deficiency not observed in the patients of the 2p21 deletion syndrome [3]. In addition, they showed a mild to moderate mental retardation and cytochrome C oxidase deficiency (mitochondrial complex IV) [4].

We have previously shown that CaM KMT is transcribed in a wide range of human tissues whereas the loss of the first exon abolished the transcription in the cells of 2p21 deletion patients [2]. Recently, we identified CaM KMT as a class I, non-SET domain calmodulin-lysine N-methyltransferase that catalyzes the formation of a trimethyllysyl residue at position 115 in calmodulin [5].

Calmodulin (CaM) is a ubiquitous calcium-binding protein that regulates a multitude of different protein targets. It is a major transducer of calcium signaling that sets the free Ca^2+^ level by binding calcium ions more rapidly than other Ca^2+^-binding proteins [6]. CaM is frequently trimethylated at Lys-115 and its methylation status changes at different developmental stages as well as in tissue specific manners that potentially modulate its actions [5].

Aiming to better characterize CaM KMT and its potential contribution to the 2p21 deletion syndrome, we demonstrate alternative transcription from the CaM KMT exons outside the 2p21 deletion syndrome, but they are not likely to be translated, the accumulation of hypomethylated calmodulin in patients compared to normal controls suggesting that CaM KMT plays a pivotal role in calmodulin methylation and also that there are no compensatory mechanisms for CaM methylation in humans. Furthermore, cellular localization studies revealed that the full-length CaM KMT localizes to the cytoplasm and nucleus in accordance with a similar subcellular distribution of calmodulin, whereas the short variant of CaM KMT (CaM KMTsh) that lacks the methyltransferase domain is localized to the Golgi complex. Western blot analysis showed expression of the CaM KMT variant in various mouse tissues. Furthermore we show that Hsp90 a highly conserved molecular chaperone [7] required for the late-stage folding of a number of classes of proteins referred to as client proteins, interacts through its middle domain with Cam KMT. Client proteins depend on Hsp90 function for the correct folding, maturation and become deregulated upon limited Hsp90 activity [8], and CaM KMT relies on Hsp90 chaperone activity for its stability since geldanamycin [9] treatment of HeLa cells transfected with CaM KMT resulted in a geldanamycin concentration dependent decrease in CaM KMT. These data indicate that CaM KMT is a novel client protein for Hsp90 and provide a new connection between Hsp90 chaperon function and CaM methylation.

## Materials and Methods

### Cell Culture

HeLa, COS-7 and HEK293 cells were maintained in Dulbecco’s modified Eagle’s medium (DMEM), supplemented with 10% fetal calf serum and 2 mM L-glutamine, 100 U/ml penicillin, 0.1 mg/ml streptomycin, 12.5 µg/ml nystatin, at 37°C in humidified atmosphere of 5% CO_2_. Lymphoblastoid cell lines from 2p21 deletion patients and normal individuals (approved by the Soroka Medical Center IRB and participants provided written informed consent) were maintained at a logarithmic growth phase in 1640 RPMI supplemented with 10% fetal calf serum, 2 mM glutamine, 100 U/ml penicillin, 0.1 mg/ml streptomycin, 12.5 µg/ml nystatin and 2.5 mg/ml Amphotericin B at 37°C in humidified atmosphere of 5% CO_2._

### Constructs

Preparation of mammalian expression vectors for CaM KMT with C-terminal GFP tag: GFP-CaM KMTsh mammalian expression vector was constructed by PCR amplification using pDNR-LIB-CaM KMTsh as a template and the following forward and reverse primers: 5′CGGAATTCA AATGGAGTCGCGAGTCG3′; 5′ACGCGTCGACCATTTTCCCCTGGTTGCTT3′ subcloned into the EcoRI and SalI sites of pEGFPN1 plasmid (Clontech). The GFP-CaM KMT full length variant was subcloned into the XhoI and EcoRI of pEGFPN1 plasmid (Clontech) by PCR amplification using pGEX-5X-1-CaM KMT construct as a template and the primers: forward-5′CTCGAGATGGAGTCGCGAGTCG3′, reverse-5′GAATTCGCTTTC CATGTTTGGTC3′. Preparation of mammalian expression vector for CaM KMT with N-terminal Myc-tag: The CaM KMT was amplified by PCR using FLAG-CaM KMT construct as a template, with the primers: forward-5′TTAGAATTCAT GGAGTCGCGAGTCGCG3′, reverse-5′TTACTCGAGCTATCCATGTT TGGTCAAAAT3′. The PCR product was subcloned into the EcoRI and XhoI sites of pCan expression plasmid. Cloning of the CaM KMT into bacterial expression vector pGEX-5X-1 with N-terminal GST tag: GST-CaM KMT was restriction digested out of pFLAG-CMV5a-CaM KMT with EcoRI enzyme and ligation into the EcoRI site of the pGEX-5X-1 plasmid. GST-CaM KMTsh was subcloned into the EcoRI and XhoI of the pGEX-5X-1 vector by PCR amplification using GFP-CaM KMTsh as a template and the primers- forward-5′GAATTCATGGAGTCGCGAGT CG3′; reverse-5′CTCGAGTCATTTTCCCCT GGTTGC3′. All constructs were verified by DNA sequencing on an ABI PRISM 3100 DNA Analyzer with the BigDye Terminator v. 1.1 Cycle Sequencing Kit according to the manufacturer’s protocol (Applied Biosystems, CA, USA). GST-Hsp90 C, M, N terminal domains were a kind gift from professor F. Ulrich Hartl, Max-Planck-Institute of Biochemistry, Munich, Germany.

### Transient Transfection

All transfections were performed using TransIT-LT1 reagent (Mirus). For Western blots and immunoprecipitation experiments, cells were plated at density of 2×10^6^ and 1×10^5^ cells per 100 mm plate and per 1 well of 6-well plate respectively, 24 hours prior to transfection. Cells were harvested 24–48 hours after transfection.

### Antibodies

The anti-Myc monoclonal, anti-FLAG monoclonal (M2), anti-GFP antibodies were purchased from Sigma-Aldrich. The anti-Hsp90 alpha/beta (F-8), anti-GAPDH antibodies were obtained from Santa Cruz Biotechnology. Peroxidase (HRP) - conjugated whole IgG secondary antibodies, and Cy3, Cy2-conjugated secondary immunofluorescence antibodies were from Jackson Immunoresearch Laboratories. Anti-CaM KMT polyclonal antibodies were raised in rabbits by immunization with a GST-CaM KMT fusion proteins followed by affinity purification. The anti-CaM antibody raised in mouse was purchased from Invitrogen. The AP-conjugated IgG secondary antibody specific for mouse was from BioRad.

### CaM KMT Polyclonal Antibody Production

CaM KMT polyclonal antibodies were generated by immunizing two New Zealand white rabbits with GST-CaM KMT fusion protein. Subcutaneous injection of 1 ml of antigen solution containing approximately 100 µg of the purified recombinant proteins, using Freund’s complete adjuvant were done for the initial immunization. The antigens were injected as native and denatured proteins to produce antibodies that would be useful for Western and immunoprecipitation experiments. Rabbit serum was collected before immunization as a negative control. Three boosts were given with intervals of 3 to 5 weeks, using Freund’s incomplete adjuvant. Serum was collected 1 week after the last injection. All blood samples were refrigerated for 16 h and centrifuged (450 × g; 10 min) at room temperature. The serum was introduced to further purification [10] and stored at −80°C.

### RNA Isolation and cDNA Preparation

Total RNA was isolated from lymphoblastoid cells using the EZ-RNA Total RNA Isolation Kit (Biological Industries). Total human RNA of different tissues was purchased from Clontech. Reverse transcription was done using Reverse-iT 1^st^ Strand Synthesis kit (ABgene). The quality of the resulting cDNA has been tested by the amplification of tubulin chaperone E gene with the primers: forward-5′AAAACGTCCATGTTCCCATC3′; reverse-5′CCCCAGACACGATAAGCAGT3′.

### RT-PCR

RT-PCR was performed on 1–3 µl of cDNA, 0.5 µM of each primer, 2.5 mM dNTP’s, Taq DNA polymerase (Fermentas). Primer in exon 11: 5′ATAAG CAGAAGCGGGTAATGA3′, primer in the new found exon: 5′GCCTCAAACTCCTGAACTGC3′, primer in exon 4 of the long isoform: 5′GCAACCATGAGCCCAGCCAAGC3′.

### RACE-PCR-SMART 5′

RACE cDNA amplification kit (Clontech) was used to amplify potential CaM KMT transcripts from lymphoblastoid cells of patients and normal controls. For the first strand synthesis we used the universal primer mix (UPM) as the forward primer and the reverse primer was designed at the border of the 5^th^ and 6^th^ exons of the long variant, to avoid priming in the residual DNA that may be retained in the RNA preparation: 5′GCACATTTCTGATGGCCTTTTCATTCC3′.The product was then amplified using nested PCR reaction with UPM and a reverse primer that was located within the 4^th^ exon of the long variant (presented in the RT-PCR paragraph). The RACE products were cloned into pGEM-T easy vector (Promega) and transformed into DH5α strain of E.coli.

### Fluorescence Imaging

Cells were grown on cover slips, fixed with freshly prepared 4% paraformaldehyde/PBS for 15 minutes, then washed extensively in PBS and mounted with Prolong Gold antifade reagent containing DAPI (Invitrogene) on microscope slides. The samples were visualized on a Leica DMR compound microscope equipped for immunofluorescence and photographed with a Spot RT digital camera (Diagnostic Instruments). Confocal fluorescent images were obtained by a Zeiss LSM Axiovert 100 laser scanning microscope.

### Whole Cell Protein Extraction

Cells were collected by scraping, pelleted by centrifugation and washed with cold PBS three times before lysis. To stabilize transient and weak protein-protein interaction, the cells used for immunoprecipitation were treated with formaldehyde (Sigma) 1% for 15 minutes and quenched with 1.25 M glycine/PBS prior to collection [11]. The cells (1×10^8^ cells) were lysed in 1 ml modified RIPA buffer (50 mM Tris HCl, pH 8.0, 150 mM NaCl, 1% NP40, 1 mM EDTA, protease inhibitors (Sigma)) for 20 min on ice, followed by centrifugation in an Eppendorf microfuge for 20 minutes at 13000 rpm at 4°C to remove insoluble debris. The supernatant was either used directly or stored at −80°C.

### Extraction of Mouse Tissues

The tissues from the ICR mice were frozen at −80°C and the lysates were prepared immediately before the Western blot experiment. The tissues were homogenized in RIPA buffer 50 mM Tris HCl, pH 8.0, 150 mM NaCl, 1% NP40, 0.5% sodium deoxycholate, 0.1% SDS, 1 mM EDTA, protease inhibitor (Sigma) using Polytron-PT-2100 homogenizer. Tissue and cell debris were removed by centrifugation at 4°C for 20 minutes, 12000 rpm. Protein concentration was determined with Bio-Rad protein assay. The lysates were boiled for 5 min in 1×SDS sample buffer (50 mM Tris-HCl pH 6.8, 12.5% glycerol, 1% SDS, 0.01% bromophenol blue, 5% β-mercaptoethanol) and 100 µg of proteins were loaded. The purified antibodies were diluted 1∶200 with 2.5% milk in TTBS, preimunne serum at 1∶50 and anti-HSP90 1:200 with 2.5% milk in TTBS. The samples with formaldehyde crosslinking were boiled for 40 minutes, before they were separated by SDS-PAGE.

### Immunoprecipitation, Western Blot Analysis and Coomassie Staining of SDS-PAGE

For immunoprecipitation, samples of 0.5–1 mg protein from formaldehyde cross linked lysates were incubated with appropriate amount of antibodies or with unrelated IgG antibody as a negative control, for 1 hour at 4°C. Then 20 µl protein A/G agarose beads (Santa Cruz) were added to each sample and incubated for 1 hour at 4°C. The beads were precipitated and washed for 10 minutes with the RIPA modified lysis buffer. Washing was repeated four times. All steps were performed with mild agitation. SDS sample buffer was added to the beads after the last wash, and then the samples were boiled, separated by SDS-PAGE and either immunoblotted with the appropriate antibodies or stained with the Coomassie blue based sensitive staining (Imperial protein stain, Pierce) according to the manufacturer’s instructions. For mass spectrometric analysis, the protein bands were excised from the stained gel and delivered to the Biological Mass Spectrometry Facility at Weizmann Institute of Science or to the University of Kentucky proteomics core facility.

For Western blot analysis, protein samples were separated on 12% SDS-polyacrylamide gels, then transferred to nitrocellulose membranes (BioTrace NT, Pall Inc.). The efficiency of transfer was monitored by Ponceau-S (Sigma) staining. The membranes were blocked for 1 h at RT with 5% milk (Sigma) in TTBS. Incubation with the primary antibodies was for 1 h at RT or overnight at 4°C. The membranes were washed three times with TTBS for 5 minutes each then incubated with secondary antibody for 1 hour at room temperature and subsequently washed with TTBS four times for 5 minutes. Blots were exposed and developed using the ECL blot detection reagent EZ-ECL (Biochemical Industries) using Chemi Doc XRS+ digital camera with Image Lab software. Western blot analyses for CaM in cell lysates were performed similarly except that PVDF membranes were used (with 20 µg of protein) and the membrane was developed using NBT/BCIP (Sigma).

### Expression and Purification of GST-Fusion Proteins

All the GST-fusion proteins were produced in Rosetta *E.Coli* cells, growing in YTx2 medium, by induction with 0.1 mM IPTG for three hours. Cells were lysed in the presence of 100 µM PMSF with seven 20-s sonicator pulses 50% duty on ice. The resulting lysate was centrifuged for 40 min at 12,000 rpm at 4°C. The proteins were then purified from the lysate by binding to glutathione-Sepharose 4B beads (Amersham Biosciences) according to the manufacturer’s instructions; the GST-fusion proteins were eluted with 30 mM glutathione, 50 mM Tris-HCl, pH 7.5, and 120 mM NaCl.

### Pull Down Assays

Lysate from HeLa cells transfected with Myc-CaM KMT or Myc plasmid containing 3 mg protein were incubated with 20 ul glutathione sepharose beads conjugated to 15 µg purified GST-Hsp90 N, M, and C-terminal fragments or GST as a negative control for overnight, at 4°C, with mild agitation. The beads were precipitated and washed four times for 10 minutes with the RIPA modified lysis buffer. Washing was repeated 4 times. Western blot was performed using anti-Myc antibody.

### CaM Methylation Assays

Cell lysates from lymphoblastoid cells (harvested as described above) were obtained by sonication in 50 mM Tris pH = 7.5, 150 mM NaCl, 5 mM DTT, 0.01% Triton X-100, 1 mM PMSF (eight 5 second pulses at 60% power on ice). The lysates were then clarified by centrifugation at 16000 g at 4°C for 10 min. The assays, in a final volume of 100 µl, contained 100 mM bicine pH 8, 150 mM KCl, 2 mM MgCl_2_, 2.5 mM MnCl_2_, 0.01% Triton X-100, 100 µM CaCl_2_, 2 mM DTT, 10 µCi [*^3^H-methyl*] AdoMet (70–80 μ Ci mmol^−1^,from PerkinElmer), 5 µg of human CaM KMT (*Hs*CaM KMT), expressed using a SUMO vector and purified according to [5], and 100 µg of total protein from cell lysates. All reactions were performed at 37°C for 2 hours and terminated by protein precipitation with 25 volumes of 10% (v/v) trichloroacetic acid. The precipitated protein pellet was dissolved in 150 µl of 0.1 N NaOH and precipitated again with the same volume of trichloroacetic acid prior being dissolved in SDS-PAGE loading buffer. The samples were electrophoresed on 12.5% SDS-PAGE gels, and transferred to a PVDF membrane prior to phosphorimage analyses.

## Results

### CaM KMT is Alternatively Transcribed in 2p21 Deletion Syndrome Patients

**Figure 1:**
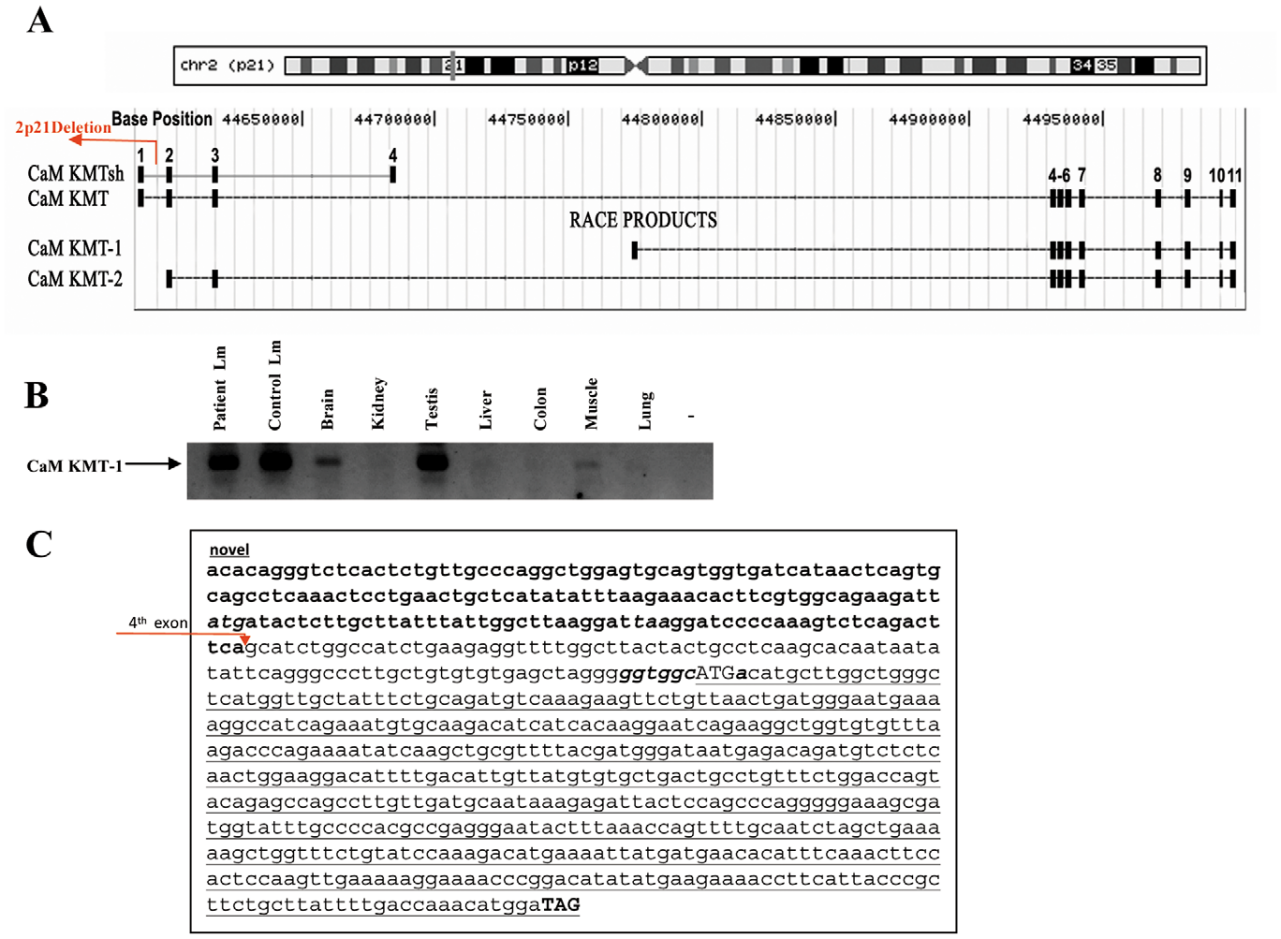
Identification of alternative *CaM KMT* variants and their expression pattern. (A) Schematic representation of the new splice variants *CaM KMT-1* and *CaM KMT-2* that were identified by 5′RACE-PCR, and their positions relative to the known full length *CaM KMT* and short *CaM KMTsh* variants. The top of the figure shows the position on chromosome 2 and the ruler of the bases according to genome assembly hg19. The site of the 2p21 deletion is marked by an arrow. (B) Verification of the transcription of the *CaM KMT-1* variant by RT-PCR in the lymphoblastoid cells from patients and controls as well as in normal human tissues. The 5′ primer was localized in the newly discovered exon and 3′ primer in 4th exon of *CaM KMT*. The identity of the products was validated by sequencing. The arrow points to the products of an expected size of 287 bp. -, no cDNA; Lm, lymphoblastoid cells. (C) The sequence of the novel mRNA *CaM KMT -1* isoform. Bold bases represent the sequence of the novel exon directly connected to the sequence of the 4th known exon that is marked by an arrow. The open reading frame is underlined and the start codon is shown in uppercase with Kozak consensus sequence shown in bold italic. The stop codon is shown in bold uppercase.

The CaM KMT gene has two splicing variants that share the first three exons (Fig. 1A). CaM KMTsh, the short variant, has a 4^th^ exon, whereas the long variant has eight additional exons and we demonstrated that it has calmodulin-lysine N-methyltransferase activity [5]. We previously reported that in accordance with deletion of all the 5′ sequence, including the promoter region, first exon and additional 300 bp into the first intron of CaM KMT in 2p21 deletion syndrome, the gene is not expressed in lymphoblastoid cells from the patients when testing with primers from the first exon. We have also demonstrated that in normal individuals both splice variants of CaM KMT have a broad transcription profile, including tissues that are affected in the 2p21 deletion syndrome: muscle, brain, testis and kidney [2]. To determine whether transcription of CaM KMT may be salvaged in the patients by the use of alternative exons outside the deletion region and more specifically in the interval of 313.9 Kb between the 3rd and 4th exons of the long isoforms we performed 5′RACE PCR on cDNA derived from patients’ lymphoblastoid cells using a primer positioned at the border of the 5th and 6th exons and a nested primer in the 4th exon of the long CaM KMT isoform. RACE-PCR products were subcloned into pGEM-T and sequenced. The results revealed two new CaM KMT splice variants derived from the patients’ cells (Fig. 1A). The first exon of the CaM KMT-1 variant is in the genomic interval between the 3rd and 4th exons (position chr2∶44776694–44776867 on hg19), its size is 174 bp and it connects to the known 4th exon (Fig. 1C). Alignment of this exon with the genomic sequence displays the AG/GT consensus for splice site at the intron–exon boundaries. To assess whether the expression of this novel isoform CaM KMT-1 is exclusive to the patients, we tested its production in lymphoblastoid cells of a normal control and in several human tissues. We performed RT-PCR with a 5′ primer in the new exon and a 3′ primer in the 4th exon of the long CaM KMT. As shown in Fig. 1B, the new variant is also expressed in the normal control lymphoblastoid cells and in brain, testis and muscle. The second new variant, termed CaM KMT-2, starts exactly at the beginning of the 2nd exon of CaM KMT and continues to the last exon of the long isoform. To verify whether these new transcripts code for proteins we searched for open reading frames (ORFs) in the newly identified isoforms and found none. Both variants encode the ORF known for CaM KMT; the first methionine is in the known 4th exon resulting in a length of 167 amino acids (Fig. 1C). However, this initiation codon is not in a good Kozak consensus sequence, missing both most important nucleotides G, after the methionine codon and A, three nucleotides before the methionine that determine the efficiency of mRNA translation [12] (Fig. 1C). These results may suggest that no additional CaM KMT protein is expected to be produced.

### The Absence of CaM KMT Causes Accumulation of Hypomethylated Calmodulin in 2p21 Deletion Syndrome Patients

**Figure 2:**
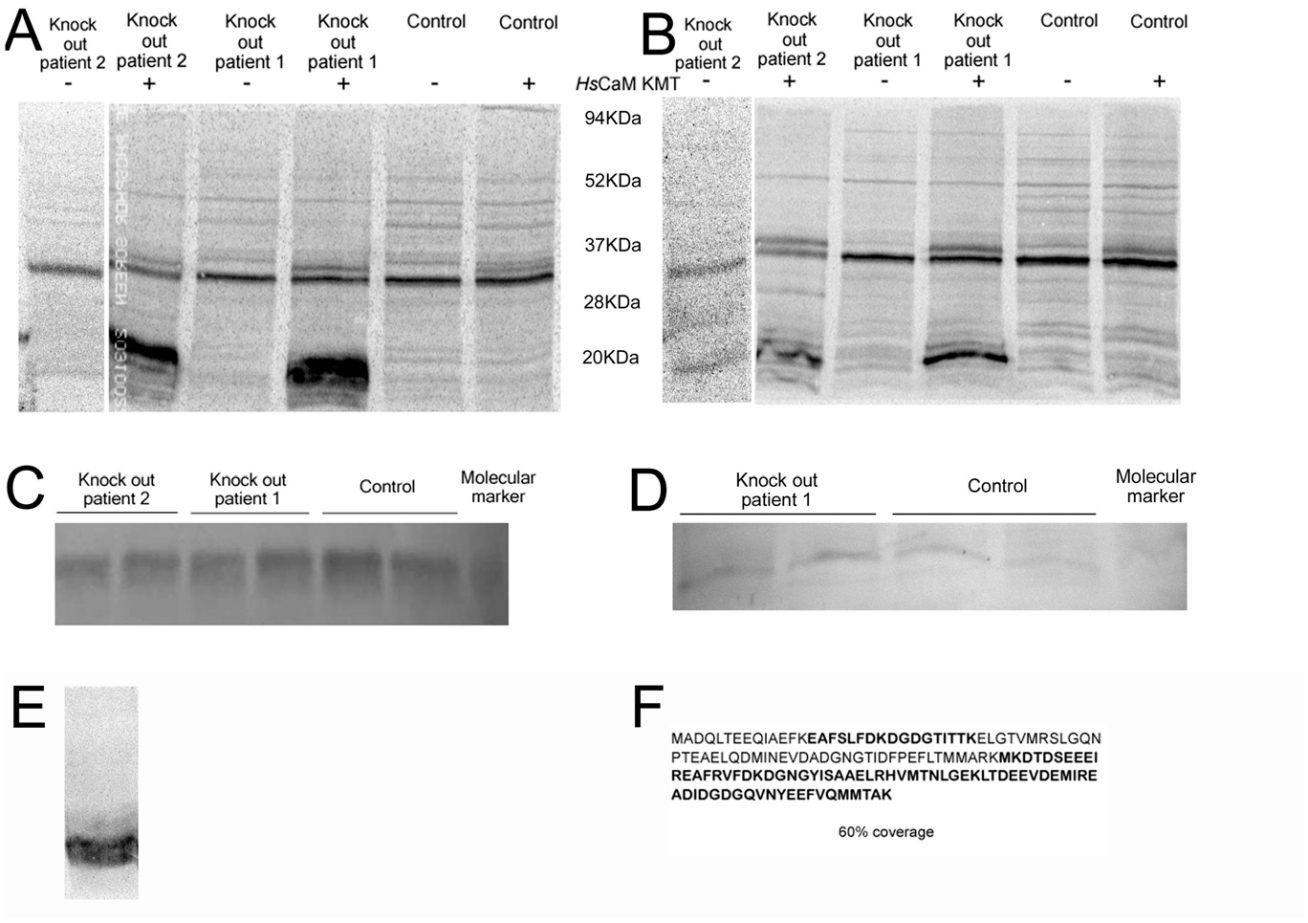
Analyses of the methylation status and relative amounts of CaM in lymphoblastoid cell lysates from a patient affected by the 2p21 deletion syndrome. (A) Phosphorimage of cell lysates from two 2p21 deletion syndrome patients and wild type individuals incubated in the presence of [*^3^H-methyl*] AdoMet with and without the addition of *Hs*CaM KMT. (B) Phosphorimage as in panel (A) but after reduction in the level of CaM by treatment of the cell lysates with phenyl sepharose. Molecular mass markers in kDa are indicated between A and B. (C) Western blot performed with anti-CaM antibody to verify the presence of similar amounts of CaM in the 2p21 deletion syndrome patient and wild type individuals. (D) Western blot performed as in panel (C) on cell lysates after partial removal of CaM with phenyl sepharose. (E) Phosphorimage showing methylation of phenyl sepharose-bound CaM removed from 2p21 deletion syndrome patients cell lysates by the addition of *Hs*CaM KMT and [*^3^H-methyl*] AdoMet. Molecular mass markers in kDa are indicated on the right. (F) Identification of the protein radiolabeled by incubation with [*^3^H-methyl*] AdoMet and *Hs*CaM KMT in panel (A) as CaM by MS/MS analysis. Peptides identified after tryptic digestion are shown in bold, approximately 60% of the entire CaM sequence was identified. The arrow indicates the position of *Hs*CaM KMT.

It has been reported that the methylation state of CaM changes in developmental and tissue dependent manners potentially affecting the interaction of CaM with target proteins, thus influencing various cellular processes [5], [13]–[15]. Since the 2p21 deletion syndrome patients do not express CaM KMT, we evaluated the methylation status of CaM in two 2p21 deletion syndrome patients’ lymphoblastoid cells. We performed an *in vitro* methylation assay using lysates from lymphoblastoid cells from patients and normal controls as a source for CaM as a substrate. The lysates were incubated with purified SUMO-*Hs*CaM KMT and [*^3^H-methyl*] AdoMet as the methyl donor. A protein of the molecular size of CaM was radioactively labeled in patient cells’ lysates, while this labeling was absent in normal controls (Fig. 2A). We confirmed that the methylation occurred on CaM and not on another cellular protein with a similar molecular mass, by depletion of the radiolabeled band by chromatography on phenyl-sepharose that binds CaM [16] (Fig. 2B), immunoblotting analysis for CaM that demonstrated comparable quantity of CaM in patients and control cells (Fig. 2C) and a reduced amount of CaM after phenyl sepharose depletion, with still comparable amount in patient and normal individual (Fig. 2D). MS/MS analysis of a non-radiolabeled immuno-reactive band from a duplicate experiment that shows 60% coverage of the polypeptide sequence for CaM including un-methylated Lys-115 from the patients’ cells is reported in Fig. 2F. Finally, to prove that CaM from patient cells could still be methylated by SUMO-*Hs*CaM KMT *in vitro*, we purified CaM from patients cells by phenyl-sepharose and then incubated it with *Hs*CaM KMT and [*^3^H-methyl*] AdoMet and a strong radiolabel incorporation was detected (Fig. 2E). An additional analysis of the methylation status of CaM in patient and normal cells was conducted by mass spectrometry on CaMs after phenyl sepharose purification. A mass of 1349Da was detected in the patient cells (fig. S1A), corresponding to peptide L116-R126, obviously a product of tryptic digestion at K115, and another peptide of 2359Da corresponding to H106-R126 without methyl groups on K115. The absence of methyl groups was also confirmed by the absence of any mass corresponding to peptide H106-R126 containing trimethyllysine. CaM from normal individual (Fig. S1B) was demonstrated to be fully methylated, presenting peptides corresponding to sequence H106-R126 containing a fully methylated K115 and different level of oxidation on methionines (peptides 2417Da and 2433Da). No peptides containing unmethylated K115 were visible (fig. S1B and S1C). These results show that the deletion of CaM KMT in patients promotes accumulation of hypomethylated CaM that can be methylated *in vitro* by *Hs*CaM KMT, and further demonstrate the absence of any compensatory cellular mechanisms for methylation of Lys-115 in CaM.

When CaM KMT was added to cell lysates in the presence of [*^3^H-methyl*] AdoMet we observed radiolabel incorporation into *Hs*CaM KMT (Fig. 2B, arrow). This may be self-methylation since incubation of GST-CaM KMT fusion protein purified from bacteria with [*^3^H-methyl*] AdoMet resulted in labeling of GST-CaM KMT (see Figure S2).

### The Subcellular Localization of the CaM KMT Proteins

**Figure 3:**
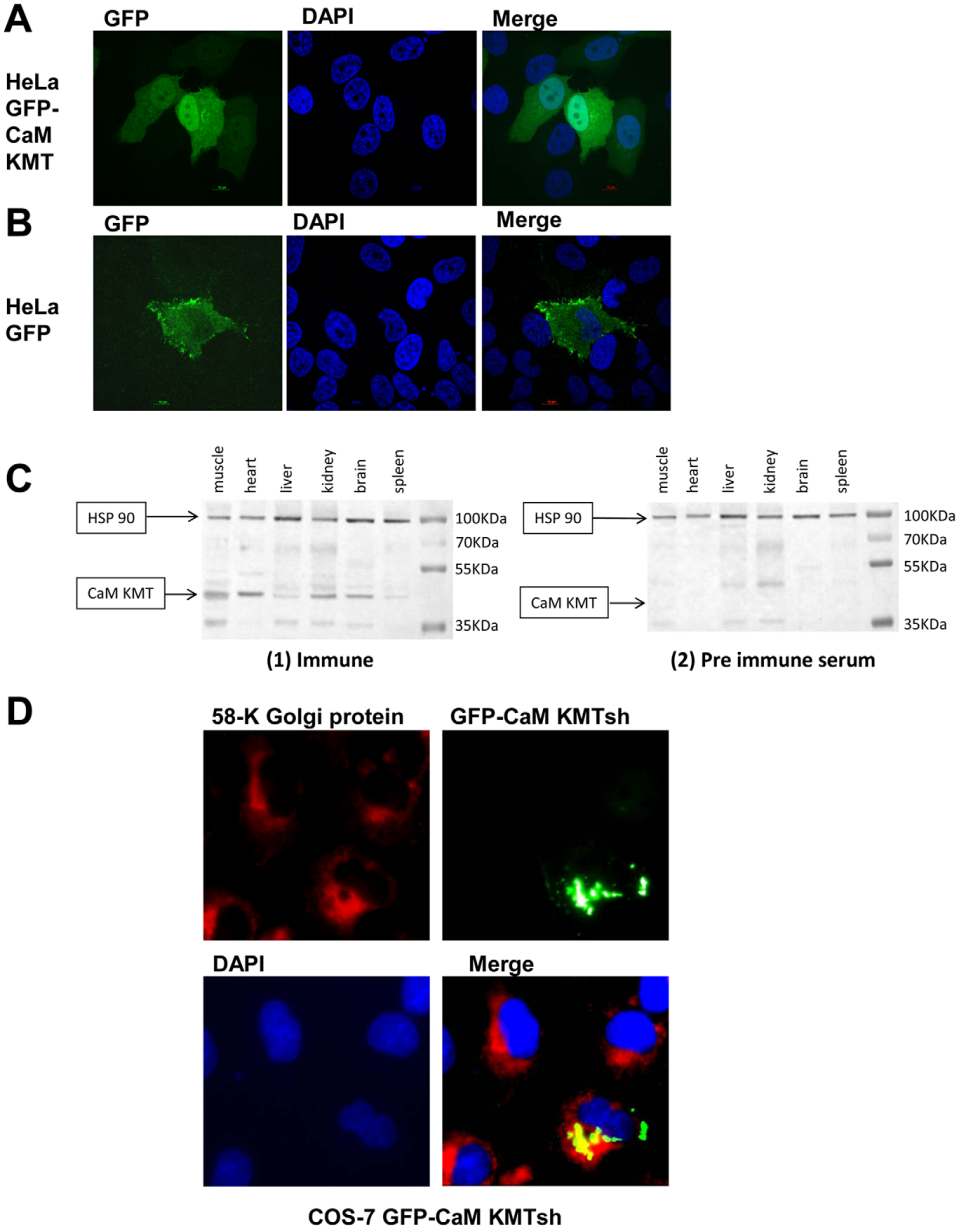
Subcellular localization of the CaM KMT-GFP fusion proteins in transiently transfected cells and expression in mouse tissues. (A) GFP- CaM KMT is localized in the cytoplasm and the nucleus. Confocal images of HeLa cells expressing CaM KMT-GFP (green), nuclear staining by DAPI (blue) and the merged image. (B) The expression of the GFP only. Confocal images of HeLa cells expressing GFP (green), staining of nuclei by DAPI (blue), and the merged image. (C) Cell lysates (100 µg of protein/lane) from mouse muscle, heart, liver, kidney, brain and spleen were resolved by SDS-PAGE, transferred to a nitrocellulose membrane, and blotted with an affinity purified polyclonal anti-CaM KMT antibody (1) immune and (2) pre-immune serum. Anti-HSP90 antibody served for protein loading control, 100 µg protein/lane were analyzed. Positions of CaM KMT and HSP-90 are indicated by the arrows. (D) GFP- CaM KMTsh is localized to the Golgi. COS-7 cells were transfected with the GFP- CaM KMT short variant and immunostained with primary antibodies against Golgi 58 k protein. GFP-CaM KMTsh was detected directly by the fluorescence microscopy (green) and 58 k Golgi protein was visualised with Cy3-labeled secondary antibodies (red). Cells nuclei were stained with DAPI (blue). Shown is the merged image presenting colocalization (in yellow) of the GFP-CaM KMT short protein with Golgi apparatus.

To determine the subcellular localization of CaM KMT we subcloned it into pEGPF-N1 expression vector, which produce CaM KMT in-fusion with the C-terminal GFP tag and studied the cellular localization by confocal microscopy. Transfection of the CaM KMT-GFP into HeLa cells, showed both cytoplasmic and nuclear localization which was distinct from the diffused cellular localization of GFP control construct (Fig. 3A, B). We concluded that CaM KMT has nuclear and cytoplasmic distribution. We also determined the subcellular localization of the short CaM KMT variant, encoding a protein of 132 amino acids. This variant contains the same three 5′ exons as CaM KMT and an additional 4th exon that lacks the methyltransferase domain. COS-7 cells were transfected with the GFP-CaM KMTsh construct and analyzed by fluorescence microscopy. GFP-CaM KMTsh overexpression revealed a discrete localization near the nucleus, similar to the Golgi apparatus localization. To verify if CaM KMTsh was sublocalized to the Golgi, COS-7 transfected cells with the GFP-CaM KMTsh were immunostained with the Golgi marker, anti-58 k antibody. The fluorescent signals from the two proteins overlapped considerably, indicating that GFP-CaM KMT could localize to the Golgi (Fig. 3D). These results suggest that the short CaM KMT variant has a distinct subcellular localization from the full length variant.

Using the affinity-purified polyclonal anti-CaM KMT antibody, we examined endogenous CaM KMT expression in different mouse tissues (Fig. 3C). Protein bands with the expected molecular masses of CaM KMT (36 kDa) were detected in most of the tissues examined, with the highest expression in the brain and muscle. The short variant could not be detected. These data support that CaM KMT is a ubiquitously expressed protein, including the high expression in the tissues affected in 2p21 deletion syndrome.

### CaM KMT Interacts with Hsp90 Molecular Chaperon

**Figure 4:**
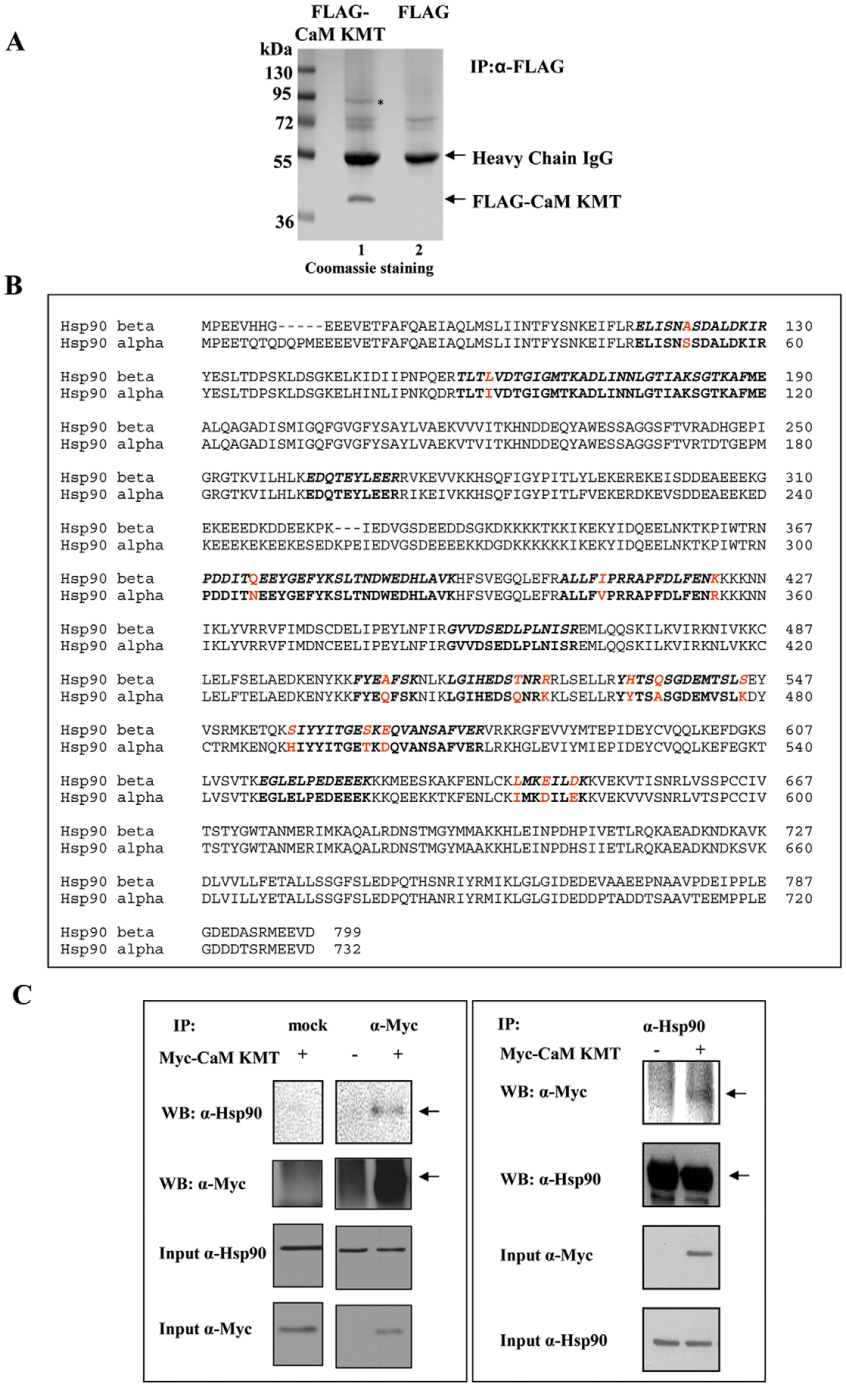
CaM KMT interacts with Hsp90. (A) Lysates of HEK293 cells transiently transfected with FLAG- CaM KMT or FLAG were immunoprecipitated with anti-FLAG antibody. The precipitated proteins were subjected to SDS-PAGE and then Coomassie stained. Molecular mass markers in kDa are indicated on the left. The band of approximately 90 kDa (shown with the asterisk) was excised from the gel, and analyzed by mass spectrometry. The heavy chains of the antibodies ∼50 kDa, two nonspecific bound proteins about 70 kDa and FLAG-CaM KMT immunoprecipitated protein were also observed. (B) Alignment of the protein sequences Hsp90α and HSP90β. The bold stretches of amino acids (26% of the protein sequence) represent peptide sequences as identified by mass spectrometry in the NCBI data bank matching Hsp90α and Hsp90β. Diverse amino acids in Hsp90α and Hsp90β, present in the sequenced peptides and enable to distinguish between the isoforms (shown in red). (C) CaM KMT and Hsp90 proteins immunoprecipitate each other.HEK293 cells were transiently transfected with Myc-CaM KMT or an empty Myc vector and 48 h after the transfection, equal protein amounts of whole cell lysates were immunoprecipitated using an anti-Myc (left), anti-Hsp90 (right) and mock IgG antibody (left) as a negative control. The immunoprecipitates were subjected to the Western blot analysis using anti-Myc and anti-Hsp90 antibody as indicated. Equal protein amounts in the immunoprecipitation assays were demonstrated by analysis of 1% input. These experiments were repeated three times with identical results.

To search for cellular proteins that specifically interact with CaM KMT, lysates of HEK293 cells expressing FLAG-CaM KMT were immunoprecipitated with anti-FLAG antibody. The immunoprecipitates were Coomassie stained and one predominant protein band of about 90 kDa that appeared to specifically co-purify with FLAG-CaM KMT could be distinguished. The other less intensive bands of ∼70 kDa were revealed as nonspecific in additional experiments (Fig. 4A). The 90 kDa band was excised from the Coomassie stained gel, subjected to mass spectrometry analysis and identified as the alpha and beta isoforms of the molecular chaperon Hsp90. The sequenced peptides represent 26% coverage of the amino acid sequences and allow differentiating between the α and β isoforms of Hsp90 (Fig. 4B) suggesting that both of them interact with CaM KMT. Human Hsp90α and Hsp90β homologs show approximately 85% identity to each other with molecular masses of 84 and 83 kDa, respectively. These homologs exhibit similar participation in multi-chaperon complexes and interact with the same substrates under normal conditions [17]. To ascertain the association between CaM KMT and Hsp90 we transiently transfected HEK293 cells with Myc-CaM KMT and performed immunoprecipitation with a monoclonal anti-Myc antibody. The immunoprecipitates were subjected to SDS-PAGE followed by immunoblotting with anti-Hsp90 α/β antibody. In agreement with the mass spectrometry results CaM KMT was found to bind Hsp90 (Fig. 4 C-left). Conversely, the transfected cell lysates were precipitated with anti-Hsp90 antibody and then probed with anti-Myc (Fig. 4 C-right). Thus, CaM KMT and Hsp90 proteins are suggested to be in a protein complex.

### CaM KMT Binds to the Middle Domain of Hsp90

**Figure 5:**
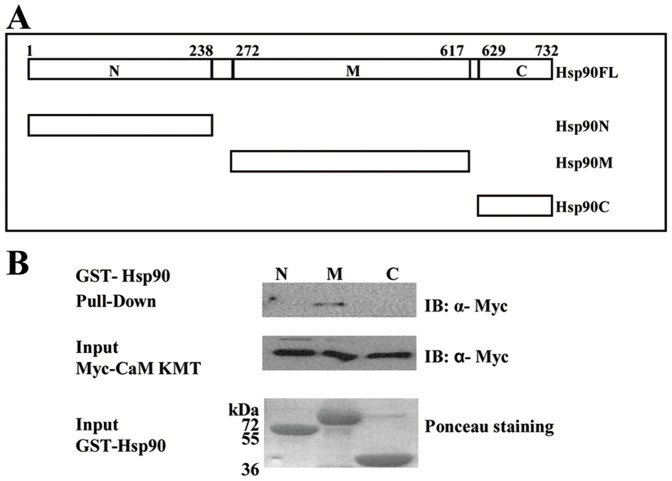
CaM KMT binds to the middle domain of Hsp90. (A) Schematic representation of the structural domains of human Hsp90 used in these experiments (numbers refer to the sequence of human Hsp90α). (B) Binding of CaM KMT and the middle domain of the Hsp90. GST fused Hsp90 fragments (10 µg protein) used in the pull-down experiments: *GST- Hsp90N* - N-terminal (9–236aa), *GST- Hsp90M* - middle domain (273–617aa), *GST- Hsp90C*- C-terminal (629–732aa), were expressed in E.Coli, purified and immobilized to glutathione agarose beads. After overnight incubation with Myc-CaM KMT transfected HEK293 cells’ lysates, the beads were washed and the bound proteins were subjected to SDS-PAGE. The membrane was stained with ponceau red before immunoblotting to demonstrate similar quantities of GST-Hsp90. Immunoblotting with anti-Myc antibody revealed that CaM KMT binds only the middle domain. Representative results from six independent experiments are shown.

Sequence alignments and proteolytic digests of Hsp90 have shown a modular structure of three domains: the N-terminal is an ATP binding domain; the C-terminal domain mediates the dimerization of the chaperons and the middle domain acts as a discriminator between different types of client and co-chaperon proteins [18], [19]. Therefore, we next asked whether the interaction between CaM KMT and Hsp90 is direct and which of the Hsp90 domains binds CaM KMT. To determine the binding site we performed pull down assays with GST constructs of each of the three Hsp90 domains. The GST-Hsp90 fragments: Hsp90N: the N-terminal domain (9–238aa), Hsp90M: the middle domain (272–617aa) and Hsp90C: C-terminal domain (629–732aa) were expressed in bacteria and affinity purified (Fig. 5A). HeLa cells were transfected with Myc-CaM KMT, lysed and then incubated with the GST-Hsp90 domains immobilized to glutathione agarose beads. Immunoblotting analysis with anti-Myc antibody demonstrated that CaM KMT binds specifically with the Hsp90 middle domain (Fig. 5B). This result indicates direct interaction of CaM KMT with the middle domain of Hsp90.

### Hsp90 is Required to Stabilize CaM KMT Protein

The Hsp90 chaperone machinery comprises numerous partner proteins: the scaffold proteins for the Hsp90 complexes and co-chaperons influence the affinity of the chaperon to substrates by regulation of ATPase cycle, recruit chaperons to specific proteins or assist in protein folding directly. Another group of Hsp90 interacting proteins is substrates or client proteins which folding, stability and conformational maturation are affected by the chaperon activity. It has been reported that several client proteins such as Akt1, Aha1, Hch1, and Src bind to the middle domain of Hsp90 whereas co-chaperons bind mostly to the C-terminal domain [20]–[23].

**Figure 6:**
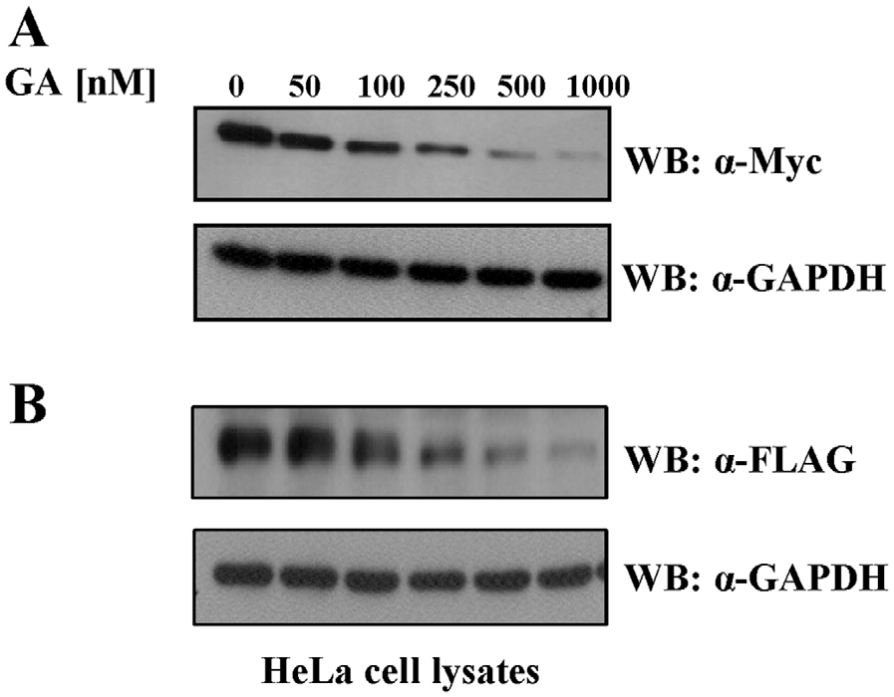
Geldanamycin induces degradation of the CaM KMT. HeLa cells transfected with Myc-CaM KMT (A) and with FLAG-CaM KMT (B) cells were treated with increasing concentrations of GA for 24 h, followed by immunoblotting with monoclonal antibodies anti-Myc (A) and anti-FLAG (B) to examine protein levels of CaM KMT. Western blot with anti-GAPDH antibody was used as proteins’ loading control. The results shown in (A) are representative of four independent experiments. The results shown in (B) are representative of two independent experiments.

Therefore, the fact that CaM KMT physically associated with the middle domain of Hsp90, encouraged us to ask whether it is a new client protein. For this purpose, we inhibited Hsp90 ATPase dependent chaperon activity with geldanamycin (GA) and tested the stability of CaM KMT. Geldanamycin is a specific antagonist of Hsp90 that binds specifically to the N-terminal ATP binding site of Hsp90, destabilizing the association between Hsp90 and its client proteins resulting in degradation of the client proteins via the proteasome pathway [24], [25]. We transiently transfected HeLa cells with Myc-CaM KMT for 24 h. At the time of the transfection we added increasing concentrations geldanamycin (GA) for 24 h. Then total cell extracts were analyzed by Western blotting with anti-Myc. As controls of equal loading of proteins we probed the membrane with anti-GAPDH antibody. As shown in Fig. 6A, GA induced a significant decline of CaM KMT protein levels in a dose-dependent manner in comparison to untreated cells. To verify that the sensitivity of CaM KMT to degradation is not tag dependent, HeLa cells were transiently transfected with CaM KMT tagged at C-terminus with FLAG tag and subsequently exposed to GA. The result demonstrated that the tag has no effect on GA induced loss of CaM KMT protein (Fig. 6B). The effect of GA was specific, since it has no effect on the GAPDH protein levels. These results suggest that CaM KMT is a novel client protein as it depends on Hsp90 chaperon activity for the stability and down regulated by Hsp90 inhibition.

## Discussion

We have previously reported an autosomal recessive 2p21 deletion syndrome in which three genes (*SLC3A1, PREPL, PP2Cβ*) and the first exon of *CaM KMT* are deleted. We demonstrated that the deletion abolished the transcript of *CaM KMT* in the 2p21 deletion syndrome patients, while the gene is ubiquitously transcribed in human normal tissues such as: brain, liver, colon, muscle and lung. The broad transcription profile of *CaM KMT* gene includes the tissues affected in the 2p21 deletion syndrome such as: muscle, brain, testis and kidney, suggesting a role for CaM KMT absence in 2p21 deletion syndrome clinical manifestations of the patients.

Here we identified two alternatively transcribed isoforms by 5′RACE-PCR experiments. These transcripts are located outside the deletion borders, thus, they are expressed in the patients’ cells as well as in several normal, human tissues. These new transcripts are not predicted to produce truncated CaM KMT proteins since they do not possess an initiator methionine codon within a good Kozak consensus sequence. However, we cannot rule out, the possibility that these transcripts could be translated since translational initiation has been shown for other proteins lacking the canonical motifs in their initiation codons [26].

We show here for the first time that loss of CaM KMT gene expression in 2p21 deletion syndrome patients results in an accumulation of hypomethylated CaM. This result proposes that CaM KMT is the major methyltransferase of CaM and there are no compensatory mechanisms for this activity in the patients. The absence of the CaM KMT activity can thus contribute to the mental retardation and mitochondrial defect observed in the 2p21 deletion patients but not in the hypotonia cystinuria patients with the absence of only the *SLC3A1, PREPL* alone. The results suggest that the methylation status of CaM may play a role in affecting CaM-dependent signaling pathways, and proteins with domains capable of reading protein methylation status have been described [27].

The importance of the methylation status of CaM has been ambiguous. The absence of methylation has been reported not to affect cell growth and viability in a chicken cell line [28]. However, the methylation status of CaM can vary in a developmentally specific manner [13], [29]. While the activity of some enzymes are directly affected by the methylation status of CaM such as plant NAD kinase [30], others like myosin light chain are not [30], [31]. Considering the relatively high number of proteins known to interact with CaM (over 300) there is likely many proteins that interact differentially with methylated versus non-methylated forms of CaM. We also noted an apparent automethylation of CaM KMT but do not know the site of methylation or whether or not it carries any biological significance. This type of autocatalytic activity has been shown for several enzymes, and it can affect different protein functions. For instance, inhibition of enzymatic activity by automethylation was identified in the DNA-cytosine-5-methyltransferase (m5C-MTase) M.BspRI [32] as well as repression for Metnase, a human SET and transposase domain protein that methylates histone H3 and promotes DNA double-strand break repair [33]. A different effect of automethylation is seen for histone H3 methyltransferase G9a. The autocatalytic G9a methylation was found to be important for protein-protein interactions. The methylation creates a binding site which mediates *in vivo* interaction with the epigenetic regulator heterochromatin protein 1 (HP1) [34], [35]. The significance of the automethylation is not known, for Dnmt3a it was suggested to be either a regulatory mechanism which could inactivate unused DNA methyltransferases in the cell, or simply be an aberrant side reaction caused by the high methyl group transfer potential of AdoMet [36].

Our analysis of the subcellular localization of *CaM KMT* within the cell showed both cytoplasmic and nuclear localization. Taking together these observations suggests CaM KMT activity probably takes place in both compartments. The distribution of CaM KMT in the nucleus and the cytoplasm seems equal in all cells, suggesting that the shuttling is not a cell cycle dependent event. However, the purpose and the mechanism of the shuttling into the nucleus remains to be further investigated. Intracellular distribution of calmodulin was also found to be both nuclear and cytoplasmic. Little is known about how the subcellular localization of calmodulin is regulated, a process that, by itself, could regulate calmodulin functions [37]. Calmodulin is the major calcium sensor in neurons when present in the cytoplasm [38]. While in the nucleus, calmodulin binds to some co-transcription factors, like BAF-57, a protein member of a complex involved in the repression of neuronal specific genes [39]. The mental retardation in the patients lacking CaM KMT may suggest an important role for CaM KMT in neuron functions. Since in the 2p21 deletion syndrome patients we previously reported reduced activity of mitochondrial respiratory complexes, except complex II [1], it was possible that CaM KMT will have a mitochondrial localization (we have tested subcellular expression of all other genes deleted in the 2p21 deletion syndrome and none localizes to the mitochondria, not reported). It could localize similar to C20orf7, a predicted methyltransferase that is essential for complex 1 assembly or maintenance and may methylate NDUFB3, complex subunit. C20orf7is peripherally associated with the matrix face of the mitochondrial inner membrane [40].

CaM KMTsh*-*GFP fusion protein was found to localize to the perinuclear structure resembling that of the Golgi complex in COS-7 and HeLa cells. However, the precise function of this variant remains obscure. The 4th exon specific to this variant is not evolutionary conserved.

Subsequent to the anti-CaM KMT polyclonal antibodies generation, the CaM KMT expression was confirmed in various mouse tissues: spleen, brain, kidney, liver, heart and muscle. These results support the suggestion that CaM KMT is a ubiquitously expressed protein, and highly expressed in the tissues affected in 2p21 deletion syndrome.

In this work we have shown that CaM KMT interacts with the Hsp90 protein. Hsp90 is a molecular chaperone and the most abundant heat shock protein under normal conditions. It exhibits ATPase activity which is essential for its chaperone function. Hsp90 binds to an array of client proteins that require its chaperon function for their folding, stabilization, ligand binding and activation. In addition, Hsp90 interacts with various types of co-chaperones, which regulate the ATPase activity of Hsp90, mediate the folding and activation of the client proteins, and direct Hsp90 to interact with specific client proteins [7]. Our mass spectrometry results have shown that both isoforms, Hsp90α and Hsp90β, interact with CaM KMT. We mapped the interaction of CaM KMT with Hsp90 to the middle domain of Hsp90, showing that it is a direct binding, by performing pull-down experiments with Hsp90 purified fragments. The middle domain is known to act as a discriminator between different types of Hsp90 interacting proteins, mostly the client proteins [21]. To verify whether CaM KMT is a co-chaperone or a client protein we inhibited the ATPase chaperon activity of Hsp90 by geldanamycin. Since the inhibition led to a significant decrease in CaM KMT protein levels we concluded that CaM KMT is a novel Hsp90 client protein. Another methyltransferase protein, Smyd3, was shown to interact with Hsp90 and this association was demonstrated to enhance the catalytic activity of Smyd3 [41]. Presumably, the biological activity of CaM KMT is also regulated by Hsp90 through a mechanism which requires CaM KMT-Hsp90 interaction. We conclude that CaM KMT is a novel, direct middle domain binding client protein and Hsp90 plays a general role in its expression levels. In this study we demonstrated that the novel CaM Lys-115 methyltransferase CaM KMT has a cytoplasmic and nuclear localization. The loss of *CaM KMT* results in hypomethylation status of CaM and we found that CaM KMT is a novel Hsp90 client protein. This study demonstrates the first step to provide basic information on *CaM KMT* that is deleted in patients of two contiguous gene deletion syndromes.

## Supporting Information

Figure S1 **Mass spectrometric analyses of CaM purified from lymphoblastoid cell lines of 2p21 deletion patients and normal individuals.** CaM purified using a phenyl sepharose resin was subjected to trypsin digestion and peptide analysis. CaM extracted from 2p21 patients (A) showed one peptide corresponding to the digested fragment L116-R126, an indication that K115 was not methylated, and one peptide corresponding to H107-R126, in which the trypsin cut was overpassed, but also shows that K115 is not methylated. The missing peptides corresponding to the same sequence H107-R126 if trimethyllysine was present are reported in between parenthesis and in a smaller font. The expected region in the spectrum where their masses should be visible are indicated by the arched shape. The analysis on CaM from normal individual (B) showed 2 peptides corresponding to the sequence H107-R126 containing one or two oxygens (indicated in figure by ox), both trimethylated at position 115 (K3Me). The peptides seen in panel A (not containing the trimethyl group on K115) were not detected in the normal individual, as also demonstrated by panel C where the peptide L116-R126 is not visible in the wild type CaM spectrum.
(TIF)

Figure S2 **Automethylation** ***in-vitro*** **of CaM KMT.** GST-CaM KMT protein (10 µg) was incubated with 5 µCi [*^3^H-methyl*] AdoMet (70–80 Ci mmol^−1^ [*^3^H-methyl*] AdoMet (from PerkinElmer), in 100 mM sodium phosphate buffer, pH 7.4, at 37°C for 1 hour. In control reaction unlabeled (cold) AdoMet (Sigma) was also added to a final concentration of 100 µM. After incubation, the reaction was terminated by the addition of SDS sample buffer, and the samples were subjected to 12% SDS-PAGE, the gel was stained with Coomassie blue staining (Imperial protein stain kit, Pierce). For fluorography, gels were treated with 2,5-diphenyloxazole (PPO) (Sigma), vacuum dried at 70°C and exposed to X-ray scientific imaging film (Kodak, MS ) at −80°C for 7–14 days.
(TIF)
