# Supplementary figures and images for: Human Calmodulin Methyltransferase: Expression, Activity on Calmodulin, and Hsp90 Dependence
Source: PLoS One. 2012 Dec 20;7(12):e52425. doi: 10.1371/journal.pone.0052425 (PMC3527508; doi:10.1371/journal.pone.0052425)

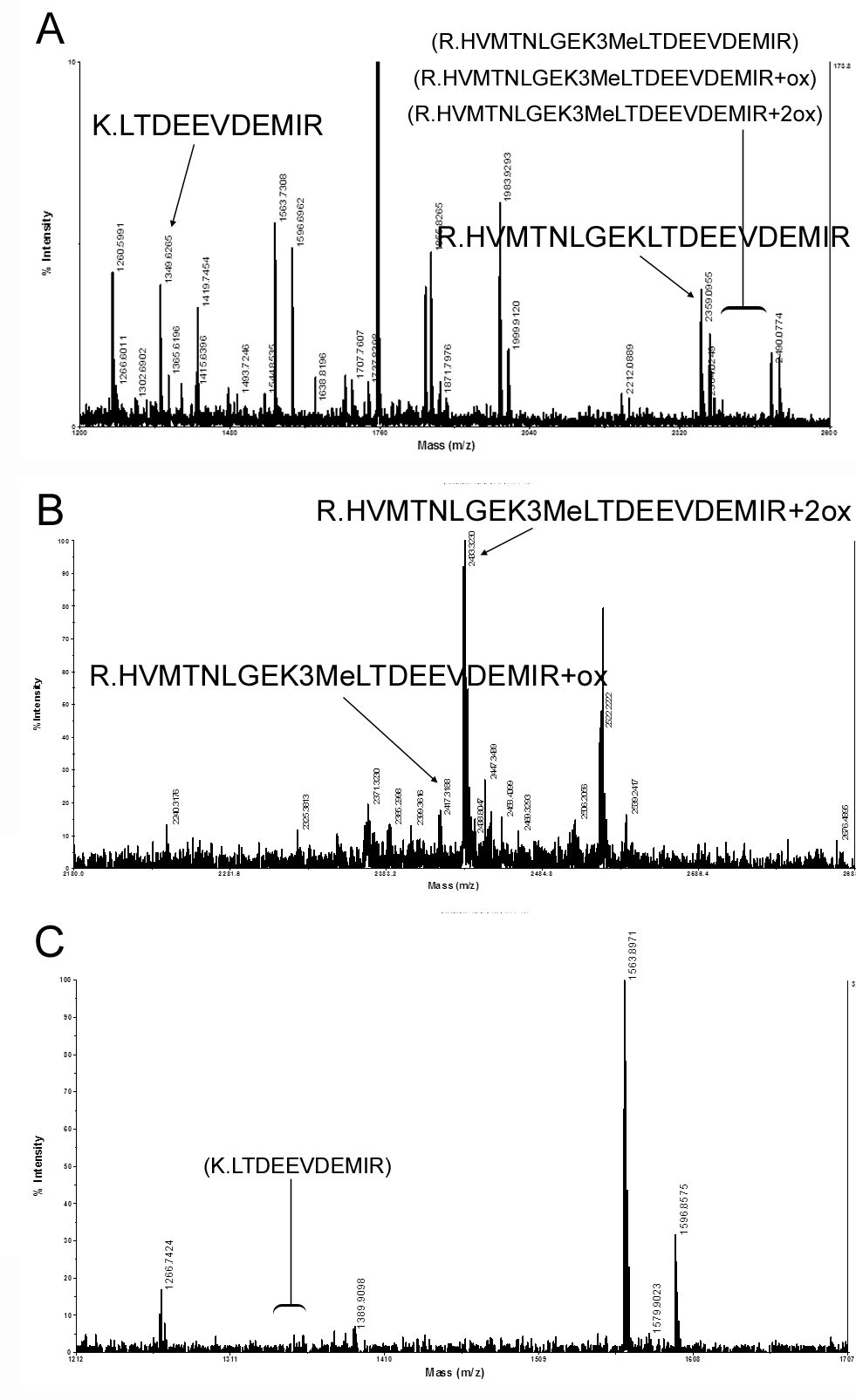

Supplement: Figure S1 — Mass spectrometric analyses of CaM purified from lymphoblastoid cell lines of 2p21 deletion patients and normal individuals. CaM purified using a phenyl sepharose resin was subjected to trypsin digestion and peptide analysis. CaM extracted from 2p21 patients (A) showed one peptide corresponding to the digested fragment L116-R126, an indication that K115 was not methylated, and one peptide corresponding to H107-R126, in which the trypsin cut was overpassed, but also shows that K115 is not methylated. The missing peptides corresponding to the same sequence H107-R126 if trimethyllysine was present are reported in between parenthesis and in a smaller font. The expected region in the spectrum where their masses should be visible are indicated by the arched shape. The analysis on CaM from normal individual (B) showed 2 peptides corresponding to the sequence H107-R126 containing one or two oxygens (indicated in figure by ox), both trimethylated at position 115 (K3Me). The peptides seen in panel A (not containing the trimethyl group on K115) were not detected in the normal individual, as also demonstrated by panel C where the peptide L116-R126 is not visible in the wild type CaM spectrum. (TIF) [file pone.0052425.s001.tif]

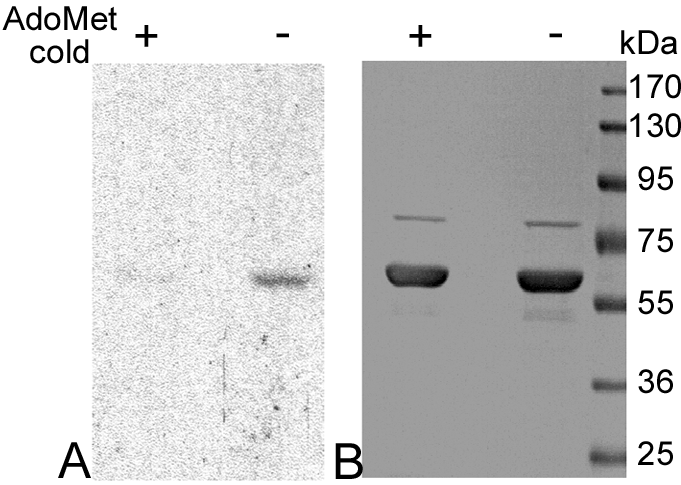

Supplement: Figure S2 — Automethylation in-vitro of CaM KMT. GST-CaM KMT protein (10 µg) was incubated with 5 µCi [3H-methyl] AdoMet (70–80 Ci mmol−1 [3H-methyl] AdoMet (from PerkinElmer), in 100 mM sodium phosphate buffer, pH 7.4, at 37°C for 1 hour. In control reaction unlabeled (cold) AdoMet (Sigma) was also added to a final concentration of 100 µM. After incubation, the reaction was terminated by the addition of SDS sample buffer, and the samples were subjected to 12% SDS-PAGE, the gel was stained with Coomassie blue staining (Imperial protein stain kit, Pierce). For fluorography, gels were treated with 2,5-diphenyloxazole (PPO) (Sigma), vacuum dried at 70°C and exposed to X-ray scientific imaging film (Kodak, MS ) at −80°C for 7–14 days. (TIF) [file pone.0052425.s002.tif]
